# A method for characterizing and analyzing the structural behavior of concrete dams in cold regions

**DOI:** 10.1038/s41598-025-32241-1

**Published:** 2025-12-19

**Authors:** Xiao Fu, Maomei Wang, Gang Zhao, Yi Xu, Yitong Qi, Chongshi Gu

**Affiliations:** 1 Jiangsu Hydraulic Research Institute, Nanjing, 210017 Jiangsu China; 2 Communication University of China Nanjing, Nanjing, 211172 Jiangsu China; 3 College of Water Conservancy and Hydropower Engineering, Hohai University, Nanjing, 210098 Jiangsu China

**Keywords:** Characterization and analysis model, Cold wave, Data processing, Freeze-thaw, Overwintering layer, Engineering, Mathematics and computing

## Abstract

Aiming at the gross error and data missing in the monitoring sequence of concrete dam, the variational mode decomposition method and the gated recurrent unit depth learning algorithm are respectively used to extract the effective information of the monitoring sequence. On the basis of the research on the characteristics of the traditional concrete dam structural behavior characterization model, the paper explores the expression mode of the effect of cold wave, freeze-thaw, wintering layer and other influencing factors. In order to reflect the correlation between the monitoring measurement values, the space coordinate variable is introduced to establish the monitoring measurement change characterization model, so as to realize the characterization and analysis of the structural behavior changes of concrete dams in cold regions and the quantitative analysis of various influencing factors. Based on the research in this article, we can fully understand the operation status of the dam, identify hidden dangers, and carry out relevant risk investigation and reinforcement. It can reduce the risk of dam failure to a certain extent.

## Introduction

Due to the distribution characteristics of hydropower resources, some dams are built in cold regions. The climate conditions in cold regions are harsh, with low ambient temperatures, severe cold waves, and frequent freeze-thaw cycles. Conventional water pressure, gravity, temperature changes and other loads, as well as harsh environmental effects, may cause material degradation and affect dam safety^1–3^. The impact of cold climate conditions and the structural characteristics of the dam itself deserve further exploration and analysis^4–6^. It is necessary to comprehensively explore the structural behavior changes of concrete dams from the perspectives of theoretical analysis, experimental research, in-situ monitoring, and numerical simulation^7–11^, which is of great significance for ensuring the safe and stable operation of the project.

The deformation behavior of the dam was firstly characterized with three parts of water pressure component, temperature component and aging component, which set a precedent for the research on the characterization model of the dam structure. Then a mixed model of dam seepage and uplift pressure considering the influence of reservoir level change in the early stage was proposed further, which improved the accuracy of the characterization model. When characterizing and analyzing the structural state of concrete dam, it is necessary to rely on effective monitoring information^12–16^. Zhang et al.^17^ proposed a missing deformation data imputation method based on bidirectional spatiotemporal imputation, which provides a reliable data foundation for comprehensive dam safety. Song et al.^18^ proposed a data-driven fusion imputation model based on novel mode decomposition and deep learning method to simulate the inherent law of dam monitoring data and predict the missing data. Deng et al.^19^ reviewed the latest progress in the field of DHM in recent years from the aspects of mathematical statistics, machine learning, monitoring equipment and so on. Due to the influence of monitoring instruments and other factors, there are gross errors and data missing in the monitoring information. The maximum posteriori probability estimation method was used to complete the data correction, which verified the effectiveness of the method in linear and nonlinear systems. Chen et al.^20^ studied the application of the ensemble empirical mode decomposition (EEMD) method in the dam deformation data, which removed the high-frequency noise and weakened the fluctuation of the measured data. With the continuous improvement of multi measuring point modeling and 3D modeling technology, many scholars have proposed numerous intelligent methods for the research on concrete dams^21–26^. Hao^27^ proposed a hybrid interval prediction model considering the volatility and nonlinearity of dam behavior sequences. Meanwhile, some scholars have conducted in-depth research on engineering projects in cold or high-altitude regions^28–31^. Zhou et al.^32^ analyzed the relationship between rock properties and concrete deterioration mode of different aggregate concrete under freeze-thaw conditions. Huang et al.^33^ conducted concrete impermeability tests on hydraulic concrete with different pore structure parameters. Li et al.^34^ studied the microstructure and mechanical properties of concrete under the combined action of different curing temperatures and freeze-thaw cycles. Lv et al.^35^ adopted a numerical simulation method that considers temperature control measures to simulate and predict the overall temperature and stress field of a certain reservoir dam. The simulation has been validated by on-site measurement results. Kang et al.^36^ simulated the effect of temperature change on the structural state of concrete dam using long-term temperature monitoring data. Huang et al.^37^ diagnosed the viscoelastic working state of dams in alpine region by considering an improved deformation monitoring model for frost heave and back analysis of dam mechanical parameters. Chen et al.^38^ combined the field test and numerical simulation methods to explore the change laws of temperature field and stress field of concrete dams in cold regions. On the basis of considering extreme climate and cold region engineering measures, Gu et al.^39^ proposed the HT-c-T model, which replaces commonly used harmonic functions or temperature with features extracted from the measured temperature field after clustering as temperature factors. This model provided an accurate and effective new method for monitoring dam displacement safety in cold regions. Yuan et al.^40^ explained the reason for the inconsistent horizontal displacement behavior of concrete dam overflow dam crest in cold regions through the proposed HTT model and numerical simulation method. Fu et al.^41^ constructed an evaluation index system affecting the structural behavior of concrete dams in cold regions, comprehensively considering the impact of overwintering layer, cold wave and freeze-thaw and the representativeness of various evaluation indexes with in-situ monitoring data. Zhao et al.^42^ proposed a method for constructing a safety risk analysis model for concrete gravity dam construction in cold regions based on fuzzy VIKOR-LEC, in order to address potential risks during the construction process.

Based on the above research, this paper proposes the gross error identification and missing value processing methods of monitoring quantity sequence, in order to extract effective information as the information basis. Then considering the effect of temperature change and freeze-thaw on the structural state of concrete dam, the construction method of characterization model is proposed after analyzing the influencing factors of monitoring measurement changes.

## Extraction of effective information for monitoring the structural behavior of concrete dams

Due to the influence of transmission noise, monitoring instrument failure and other factors, the monitoring data sequence of concrete dam in cold regions is prone to problems such as gross error and data missing. Therefore, in the process of establishing the monitoring quantity characterization model, gross error identification and missing value processing are required for the monitoring data sequence to extract its effective monitoring information.

### Gross error identification method of monitoring data sequence

#### Variational modal decomposition method for monitoring data sequence

The measurement sequence of structural state monitoring of concrete dams has typical nonlinear and non-stationary characteristics, and there will inevitably be gross errors in monitoring. Based on the idea of ‘decomposition-description-reconstruction’, this section decomposes the original sequence into high and low frequency components. The low frequency components reflect the regular changes of water pressure, temperature and aging components, while the high frequency components may have abnormal values or monitoring gross errors. The gross errors are identified and eliminated for the high frequency components, so as to reconstruct each sub sequence and realize the extraction of effective information of the monitoring sequence.

At present, some scholars have proposed wavelet analysis, empirical mode decomposition (EMD), ensemble empirical mode decomposition (EEMD) and other methods, which have achieved certain results in solving the above problems. However, wavelet analysis does not have the characteristics of self-adaptation, and it is difficult to achieve the global optimal solution by selecting wavelet functions and decomposition scales empirically. The number of decompositions in EMD or EEMD methods is random and uncontrollable, requiring multiple iterations to calculate the average value. These methods are prone to modal aliasing, which means that the IMF will contain characteristic components at different time scales. In this section, the monitoring data sequence decomposition process is transformed into a variational solution problem. Based on the variational modal decomposition method, the adaptive decomposition of data signals is realized through a non-recursive variational mode to solve the problems of modal aliasing and end effect.

Variational Mode Decomposition (VMD) decomposes complex original monitoring sequence signals into *K *(specific value of K can be determined by using the center frequency observation method, information criterion method, spectrum analysis method, cross-validation method and empirical method) intrinsic mode functions (IMF), and obtains their mode functions by determining the center frequency ${\omega _k}$ and bandwidth ${u_k}\left( t \right)$ of each natural mode decomposition, namely:

1 $${u_k}\left( t \right)={A_k}\left( t \right)\cos \left( {{\varphi _k}\left( t \right)} \right)$$

where ${A_k}\left( t \right)$ is the instantaneous amplitude of modal component ${u_k}\left( t \right)$,${A_k}\left( t \right) \geqslant 0$; ${\varphi _k}\left( t \right)$ is the instantaneous phase of modal component ${u_k}\left( t \right)$.

VMD algorithm assumes that all components are narrowband signals concentrated near their respective center frequencies. By constructing and solving the optimal solution problem of constrained variation, including equality constraints and inequality constraints, that is, the sum of the decomposed components is equal to the source signal, and the sum of the estimated bandwidth of each component is the smallest, namely:

2 $$\left\{ \begin{gathered} \mathop {\hbox{min} }\limits_{{\left\{ {{u_k}} \right\},\left\{ {{\omega _k}} \right\}}} \left\{ {\sum\limits_{{k=1}}^{K} {\left\| {{\partial _t}\left[ {\left( {\delta \left( t \right)+\frac{j}{{\pi t}}} \right) \otimes {u_k}\left( t \right)} \right]{e^{ - j{\omega _k}t}}} \right\|} _{2}^{2}} \right\} \\ s.t.\sum\limits_{k} {{u_k}} =f \\ \end{gathered} \right.$$

where ${u_k}=\left\{ {{u_1},{u_2}, \cdots ,{u_k}} \right\}$ is the decomposition components; ${\omega _k}=\left\{ {{\omega _1},{\omega _2}, \cdots ,{\omega _k}} \right\}$ is the center frequency; $\delta \left( t \right)$ is Dirac function; $\otimes$ is the convolution operator; *j* is an imaginary symbol; $s.t.$ is the abbreviation for ‘subject to’; *f* is the source signal.

In order to obtain the optimal solution of the above equation, a quadratic penalty factor and an augmented Lagrange function are introduced to convert the constrained variational problem into an unconstrained variational problem, namely:

3 $$\begin{aligned} L\left( {\left\{ {u_{k} } \right\},\left\{ {\omega _{k} } \right\},\lambda } \right) & = \alpha \sum\limits_{k} {\left\| {\partial _{t} \left[ {\left( {\delta \left( t \right) + \frac{j}{{\pi t}}} \right) \otimes u_{k} \left( t \right)} \right]e^{{ - j\omega _{k} t}} } \right\|} _{2}^{2} \, \\ & + \left\| {f\left( t \right) - \sum\limits_{k} {u_{k} \left( t \right)} } \right\|_{2}^{2} + \left\langle {\lambda \left( t \right),f\left( t \right) - \sum\limits_{k} {u_{k} \left( t \right)} } \right\rangle \\ \end{aligned}$$

where $\alpha$ is the secondary penalty factor, the larger the value, the smaller the frequency bandwidth of each component; $\lambda$ is Lagrange operator; $\left\langle {} \right\rangle$ represents the inner product operation.

The multiplier alternating direction algorithm is used to update $u_{k} ,\,\omega _{k} ,\,\lambda$ iteratively and solve it circularly. When updating one of the variables, the other two variables are fixed.

Firstly, the update iteration process of ${u_k}$ is:

4 $$u_{k}^{{n+1}}=\mathop {\arg \hbox{min} }\limits_{{{u_k}}} L\left( {\left\{ {u_{{i<k}}^{{n+1}}} \right\},\left\{ {u_{{i>k}}^{{n+1}}} \right\},\left\{ {\omega _{i}^{n}} \right\},{\lambda ^n}} \right)$$

Take one term from the first term of the augmented Lagrange function:

5 $$\begin{aligned} & \alpha \left\| {\partial _{t} \left[ {\left( {\delta \left( t \right) + \frac{j}{{\pi t}}} \right) \otimes u_{k} \left( t \right)} \right]e^{{ - j\omega _{k} t}} } \right\|_{2}^{2} + \left\| {f\left( t \right) - \sum\limits_{k} {u_{k} \left( t \right)} } \right\|_{2}^{2} \\ & + 2 \times \frac{1}{2}\left\langle {\lambda \left( t \right),f\left( t \right) - \sum\limits_{k} {u_{k} \left( t \right)} } \right\rangle + \alpha \sum\limits_{{i \ne k}} {\left\| {\partial _{t} \left[ {\left( {\delta \left( t \right) + \frac{j}{{\pi t}}} \right) \otimes u_{k} \left( t \right)} \right]e^{{ - j\omega _{k} t}} } \right\|} _{2}^{2} \\ \end{aligned}$$

Combine some terms of the above formula into a completely flat way, that is:

6 $$\begin{gathered} \alpha \left\| {{\partial _t}\left[ {\left( {\delta \left( t \right)+\frac{j}{{\pi t}}} \right) \otimes {u_k}\left( t \right)} \right]{e^{ - j{\omega _k}t}}} \right\|_{2}^{2}+\left\| {f\left( t \right) - \sum\limits_{k} {{u_k}\left( t \right)} +\frac{{\lambda \left( t \right)}}{2}} \right\|_{2}^{2} \hfill \\ +\alpha \sum\limits_{{i \ne k}} {\left\| {{\partial _t}\left[ {\left( {\delta \left( t \right)+\frac{j}{{\pi t}}} \right) \otimes {u_k}\left( t \right)} \right]{e^{ - j{\omega _k}t}}} \right\|} _{2}^{2} - \left\| {\frac{{\lambda \left( t \right)}}{2}} \right\|_{2}^{2} \hfill \\ \end{gathered}$$

Ignoring the last two smaller items, we get:

7 $$u_{k}^{{n+1}}=\mathop {\arg \hbox{min} }\limits_{{{u_k}}} \left\{ {\alpha \left\| {{\partial _t}\left[ {\left( {\delta \left( t \right)+\frac{j}{{\pi t}}} \right) \otimes {u_k}\left( t \right)} \right]{e^{ - j{\omega _k}t}}} \right\|_{2}^{2}+\left\| {f\left( t \right) - \sum\limits_{k} {{u_k}\left( t \right)} +\frac{{\lambda \left( t \right)}}{2}} \right\|_{2}^{2}} \right\}$$

Based on Fourier transform, expand $u_{k}^{{n+1}}$, convert the time domain into frequency domain according to Parseval’s Law, and update ${u_k}$ further:

8 $$\hat {u}_{k}^{{n+1}}\left( \omega \right)=\frac{{\hat {f}\left( \omega \right) - \sum\limits_{{i<k}} {\hat {u}_{i}^{{n+1}}\left( \omega \right)} - \sum\limits_{{i>k}} {\hat {u}_{i}^{n}\left( \omega \right)} +\frac{{\hat {\lambda }_{k}^{n}\left( \omega \right)}}{2}}}{{1+2\alpha {{\left( {\omega - \omega _{k}^{n}} \right)}^2}}}$$

where $\hat {u}_{k}^{{n+1}}\left( \omega \right)$ is Wiener filtering of current data signal; $\wedge$ represents Fourier transform.

The update iteration process of ${\omega _k}$ is:

9 $$\omega _{k}^{{n+1}}=\mathop {\arg \hbox{min} }\limits_{{{\omega _k}}} L\left( {\left\{ {u_{k}^{n}} \right\},\left\{ {\omega _{{i<k}}^{{n+1}}} \right\},\left\{ {\omega _{{i>k}}^{{n+1}}} \right\},{\lambda ^n}} \right)$$

Similarly, ${\omega _k}$ is transformed to the frequency domain for solution, and the update formula of ${\omega _k}$ is obtained as follows:

10 $$\omega _{k}^{{n+1}}=\frac{{\int_{0}^{\infty } {\omega \left| {u_{k}^{n}\left( \omega \right)} \right|} d\omega }}{{\int_{0}^{\infty } {\left| {u_{k}^{n}\left( \omega \right)} \right|} d\omega }}$$

Finally, according to the dual rising principle, the update process of $\lambda$ is as follows:

11 $${\lambda ^{n+1}}={\lambda ^n}+\tau \left[ {f - \sum\limits_{k} {u_{k}^{{n+1}}} } \right]$$

where $f - \sum\limits_{k} {u_{k}^{{n+1}}}$ is the gradient of dual function; $\tau$ is the fidelity coefficient. Similarly, after Fourier transform, we get:

12 $${\hat {\lambda }^{n+1}}\left( \omega \right)={\hat {\lambda }^n}\left( \omega \right)+\tau \left[ {\hat {f}\left( \omega \right) - \sum\limits_{k} {\hat {u}_{k}^{{n+1}}\left( \omega \right)} } \right]$$

The solution end condition of the iterative loop is:

13 $$\sum\limits_{k} {\frac{{\left\| {\hat {u}_{i}^{{n+1}}\left( \omega \right) - \hat {u}_{i}^{n}\left( \omega \right)} \right\|_{2}^{2}}}{{\left\| {\hat {u}_{i}^{n}\left( \omega \right)} \right\|_{2}^{2}}}} <\varepsilon$$

where $\varepsilon$ is the input threshold.

After finding the saddle point of the unconstrained variational problem, *K* modal components are obtained according to the cyclic iteration results. On this basis, the low-frequency components are removed from the sub-sequences, and the high-frequency components are separated. The high-frequency component sequence is used as the data samples for gross error retrieval and identification.

#### Gross error identification steps of monitoring data sequence

After decomposing the monitoring data sequence of concrete dams in cold regions by using the variational modal decomposition method, gross errors are identified and eliminated for the high-frequency component sequence obtained from the decomposition. Each modal component is superimposed and reconstructed to obtain the monitoring data sequence after gross error processing, so as to realize the extraction of effective information.

Taking the high-frequency component sequence $\left\{ {{x_1},{x_2}, \cdots ,{x_n}} \right\}$ of monitoring data as the calculation samples, the standard deviation ${\sigma _n}$ of the samples reflects the dispersion degree of the sequence, and the existence of individual gross errors will undoubtedly increase the value of the standard deviation of the whole sequence. After eliminating the gross error, the standard deviation of the sequence will be significantly reduced. Based on this principle, gross error identification of high-frequency component sequences is carried out. Specific implementation steps are as below:

1. Firstly, the measuring data sequence of a concrete dam is decomposed into a variational mode, and the high-frequency component sequence $\left\{ {{x_1},{x_2}, \cdots ,{x_n}} \right\}$ and its corresponding standard ${\sigma _n}$ deviation are obtained.
2. Starting from the first value of the high-frequency component sequence, remove the $k - th$ measured value in turn, calculate the posterior standard deviation ${\sigma _{n - 1,k}}$ of the remaining $n - 1$ high-frequency component sequences and obtain *n* posterior standard deviations $\left\{ {{\sigma _{n - 1,1}}, \cdots ,{\sigma _{n - 1,k}}, \cdots {\sigma _{n - 1,n}}} \right\}$ after the global elimination search.
3. If the suspected value of gross error ${x_{{m_1}}}$ makes the largest contribution to the standard deviation ${\sigma _n}$ of the original high-frequency sequence, the posterior standard deviation ${\sigma _{n - 1,{m_1}}}$ after removing the suspected value of gross error ${x_{{m_1}}}$ will be the smallest, namely:
  14 $${\sigma _{n - 1,{m_1}}}{\mathrm{=}}\hbox{min} \left\{ {{\sigma _{n - 1,1}}, \cdots ,{\sigma _{n - 1,k}}, \cdots {\sigma _{n - 1,n}}} \right\}$$
  Therefore, it can be judged that the suspected gross error value ${x_{{m_1}}}$ is an abnormal value or gross error value caused by the load condition.
4. If the above suspected value ${x_{{m_1}}}$ is gross error, after removing the value, calculate the standard deviation of the remaining $n - 1$ high-frequency component sequence $\left\{ {{x_1}, \cdots ,{x_{{m_1} - 1}},{x_{{m_1}+1}}, \cdots ,{x_n}} \right\}$as ${\sigma _{n - 1}}$, repeat the removal operation in sequence in step (3), and calculate the minimum value ${\sigma _{n - 2,{m_2}}}$ of the posterior standard deviation.
5. After M rounds of search and elimination, the posterior standard deviation and the original standard deviation of M groups $\left\{ {\left( {{\sigma _{n - 1,{m_1}}}{\mathrm{,}}{\sigma _n}} \right){\mathrm{,}}\left( {{\sigma _{n - 2,{m_2}}}{\mathrm{,}}{\sigma _{n - 1}}} \right){\mathrm{,}} \cdots \left( {{\sigma _{n - M,{m_M}}}{\mathrm{,}}{\sigma _{n - M+1}}} \right)} \right\}$ are obtained.
6. Carry out the F test on the above equation, define the ratio of the posterior standard deviation excluding the suspected gross error ${x_{{m_i}}}$ and the original standard deviation as the statistical inspection quantity ${\gamma _i}$, and judge whether the suspected gross error value ${x_{{m_i}}}$ of the above sequence is a gross error, namely:
  15 $${\gamma _i}=\frac{{{\sigma _{n - i,{m_i}}}}}{{{\sigma _{n - i+1}}}},i=1,2, \cdots ,i \leqslant M<n$$
  where ${\sigma _{n - i+1}}$ is the sequence standard deviation after *i* gross error eliminations; ${\sigma _{n - i,{m_i}}}$ is the posterior standard deviation of removing ${x_{{m_i}}}$ after *i* gross error eliminations.
7. Under the condition of given significance level $\alpha$, ${\gamma _i}$ obeys the F distribution. Judge whether to accept the hypothesis according to the critical value ${F_\alpha }$. The F test hypothesis condition is: ${\gamma _i} \geqslant {F_\alpha }$. After removing ${x_{{m_i}}}$ from the high-frequency component sequence of concrete dam monitoring data, the standard deviation of the sequence changes significantly, indicating that ${x_{{m_i}}}$ is a gross error, which should be eliminated. Before elimination, it is necessary to verify whether there is abnormal environment or load change at ${x_{{m_i}}}$, and carry out the next round of search. If ${\gamma _i}<{F_\alpha }$, it indicates that the standard deviation of the high-frequency component sequence has not changed significantly after removing ${x_{{m_i}}}$. Therefore, ${x_{{m_i}}}$ is the true value of the measuring data, and the gross error identification process ends.

After the gross error identification of high-frequency component sequence is completed, each gross error identified is processed by using the elimination method, and each modal component is superposed and reconstructed to obtain the monitoring data sequence after gross error processing. Then the effective information of the monitoring data can be obtained.

### Missing value processing method of monitoring data sequence

In addition to gross errors, there may also be missing data in the monitoring data sequence. The lack of key data will lead to misjudgment of monitoring data characteristics, which will bring difficulties to the construction of the characterization model. The commonly used interpolation methods have a good effect when there are few missing data or the degree of dispersion is high, but they are not effective when dealing with continuous missing monitoring data. Therefore, based on the deep learning algorithm of gated recurrent unit, this section explores the processing method of missing monitoring data of concrete dam in cold regions, which can provide information basis for constructing the characterization model.

**Fig. 1:**
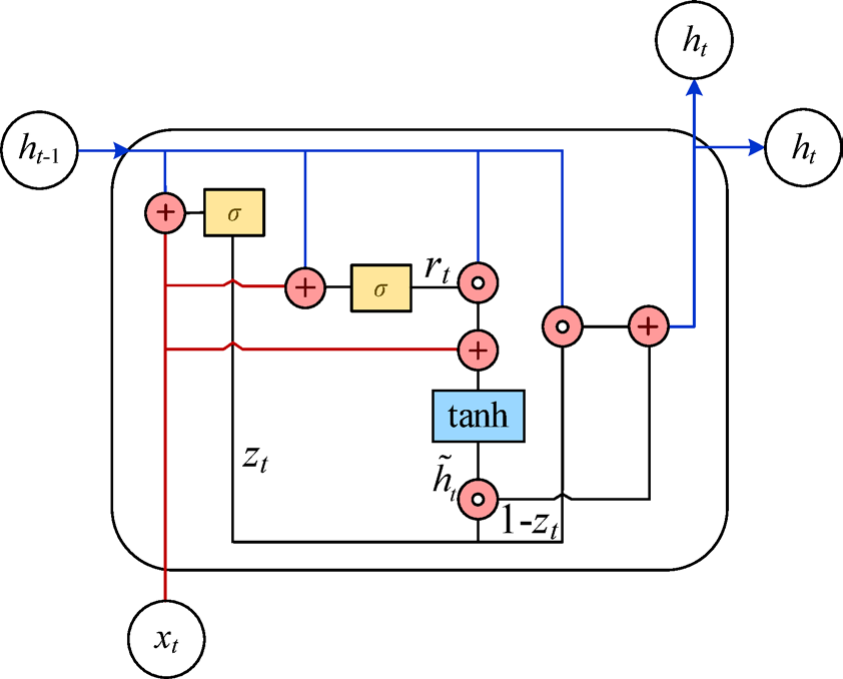
GRU unit structure diagram.

Gated Recurrent Unit (GRU) neural network is a special type of Recurrent Neural Networks (RNN), which uses a gating mechanism to control the input, memory, output and other information of the monitoring data. Compared with the standard RNN, GRU describes the long and short term temporal correlations of sequences more completely. Compared with the long short-term memory (LSTM) neural network, GRU has fewer parameters and is not prone to over fitting problems. Its network structure is shown in Fig. 1.

In Fig. 1, ${x_t}$ represents the input state at the moment *t* in the GRU network; ${h_{t - 1}}$ indicates the hidden status of the upper layer cell; ${z_t}$ represents the update gate, which determines the amount of information transferred from the hidden state ${h_{t - 1}}$ of the previous layer to the current hidden state ${h_t}$; ${r_t}$ means reset gate, which determines how much information has been forgotten in the past; $\sigma$ and tanh represent sigmoid activation function and tanh activation function respectively, namely:

16 $$\begin{gathered} {\mathrm{sigmoid}}\left( x \right)=\frac{1}{{1+{e^{ - x}}}}; \\ \tanh \left( x \right)=\frac{{{e^x} - {e^{ - x}}}}{{{e^x}+{e^{ - x}}}}; \\ \end{gathered}$$

Based on GRU neural network deep learning algorithm, the work flow of missing data processing in concrete dam monitoring is as follows:

1. Standardize the original monitoring data sequence as the training sample of GRU neural network model.
2. At a certain moment *t*, through updating gate ${z_t}$ and resetting gate ${r_t}$, the current monitoring data input state ${x_t}$ and the unit hidden state ${h_{t - 1}}$ at the previous moment are calculated. Furthermore, through matrix operation, GRU determines whether the node is activated by the activation function $\sigma$, namely:
  17 $${z_t}=\sigma \left( {{W^{\left( z \right)}}{x_t}+{U^{\left( z \right)}}{h_{t - 1}}} \right)$$
  18 $${r_t}=\sigma \left( {{W^{\left( r \right)}}{x_t}+{U^{\left( r \right)}}{h_{t - 1}}} \right)$$
  where ${W^{\left( z \right)}}$ and ${W^{\left( r \right)}}$ are input weight matrix; ${U^{\left( z \right)}}$ and ${U^{\left( r \right)}}$ are cyclic weight matrix.
3. For the current input state ${x_t}$ and the past relevant information stored through the reset gate ${r_t}$, perform linear transformation and superposition respectively. The hyperbolic tangent activation function tanh is used to form the current memory information ${\tilde {h}_t}$, namely:
  19 $${\tilde {h}_t}=\tanh \left( {W{x_t}+{r_t} \circ U{h_{t - 1}}} \right)$$
  where $\circ$ is Hadamard product.
4. By updating the gate ${z_t}$, dynamically regulate the hidden state ${h_{t - 1}}$ at the last time and the current memory information ${\tilde {h}_t}$, and determine the current moment output information ${h_t}$ of the unit:
  20 $${h_t}={z_t} \circ {h_{t - 1}}+\left( {1 - {z_t}} \right) \circ {\tilde {h}_t}$$

where the two items respectively represent the information that needs to be retained at the current moment from the previous moment and the information that needs to be forgotten at the decision.

According to the above workflow, the monitoring measurement sequence is trained for many times to obtain the trained GRU neural network model, and then the estimation value of the missing part is predicted.

Therefore, based on the research contents in Sect. 2.1 to 2.2, the variational mode decomposition method is introduced to identify and eliminate the gross errors of the monitoring data sequence, and the GRU neural network deep learning algorithm is used to process the missing data, so as to extract effective information and provide information basis for the construction of the monitoring data characterization model.

## Influencing factors of structural state monitoring of concrete dams in cold regions

In order to understand the structural behavior changes of various parts of concrete dams in cold regions, it is necessary to solve the problem of monitoring and measurement characterization of each measuring point reflecting the structural state changes of dams. In this section, according to the operating characteristics of concrete dams in cold regions, the expressions of various influencing factors are explored, which can provide a basis for the measuring data characterization model construction.

The monitoring and measurement changes of concrete dams in cold regions are mainly affected by water pressure, temperature, aging, freeze-thaw, wintering layers, etc. The deformation is analyzed as an example below. As for the deformation, it is mainly divided into five components according to its causes: water pressure component ${f_H}$, temperature component${f_T}$, aging component ${f_\theta }$, freeze-thaw component ${f_I}$ and wintering layer component ${f_Y}$. The expressions of each component are discussed below.

### Water pressure component ${f_H}$

Under the action of water pressure load, the deformation of concrete dam mainly consists of three parts: the dam deformation caused by the internal force generated by the hydrostatic pressure in the dam body, the deformation caused by the internal force generated on the foundation surface and the deformation caused by the foundation rotation. Based on material mechanics and engineering experience, the water pressure component of deformation ${f_H}$ is a high-order function relationship with upstream water depth, which is expressed as:

21 $${f_H}=\mathop \sum \limits_{{i=1}}^{{{m_1}}} {a_i}{H^i}$$

where *H* is the upstream water depth; ${a_i}$ is the water pressure factor coefficient; ${m_1}$ is taken as 3 for a gravity dam, while it is taken as 4 for an arch dam.

### Temperature component ${f_T}$

The deformation changes of concrete dams in cold regions are greatly affected by temperature change. The upstream surface of the dam body is mainly in contact with water, and the downstream surface is mainly in contact with air. When there are enough thermometers inside and on the boundary of the dam body, by subtracting the initial temperature field of the first measurement day from the instantaneous temperature field of the monitoring day, the temperature change value of each point can be obtained as follows:

22 $$\Delta {T_i}\left( {x,y,z} \right)\left| {_{{{t_i} - {t_0}}}} \right.={T_i}\left( {x,y,z,{t_i}} \right) - {T_i}\left( {x,y,z,{t_0}} \right)$$

For the convenience of analysis, $\Delta {T_i}\left( {x,y,z} \right)\left| {_{{{t_i} - {t_0}}}} \right.$ is indicated by ${T_i}$. The temperature change value of each temperature measuring point is used as a factor affecting the temperature component. Then, the temperature component is expressed as:

23 $${f_T}=\sum\limits_{{i=1}}^{m} {{b_i}{T_i}}$$

where${b_i}$ is the coefficient of the *i*-th thermometer.

**Fig. 2:**
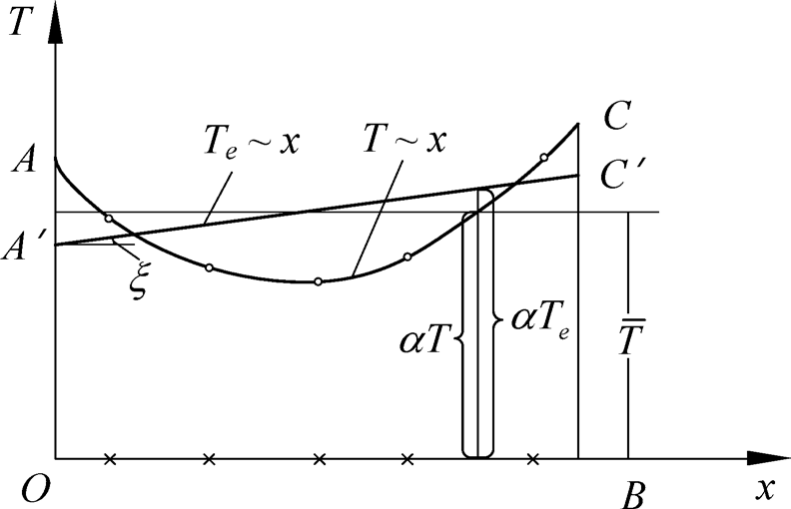
Equivalent temperature diagram of concrete dams.

When there are too many thermometers in the dam, the calculation of equivalent temperature is feasible. The average temperature change $\bar {T}$ and temperature gradient ${\beta _i}$ of the equivalent temperature are taken as the factors for modeling, as shown in Fig. 2. At the time, the temperature component is expressed as:

24 $${f_T}=\mathop \sum \limits_{{i=1}}^{{{m_2}}} {b_{1i}}{\bar {T}_i}+\mathop \sum \limits_{{i=1}}^{{{m_3}}} {b_{2i}}{\beta _i}$$

In Fig. 2 and Eq. (24), *T* is the actual temperature change distribution, ${T_e}$ is the equivalent temperature change distribution.$\bar {T}=\frac{{{A_t}}}{B}$,${A_t}$is the actual distribution area of temperature variation, *B* is the cross-sectional width; $\beta =\frac{{12{M_t} - 6{A_t}B}}{{{B^3}}}$,${M_t}$ is the area moment of ${A_t}$ about the OT axis; ${b_{1i}}$and ${b_{2i}}$ are the regression coefficients.

When the monitoring data of the thermometer embedded in the dam body is insufficient, the hydration heat inside the concrete dam during operation is basically distributed, and the internal temperature field is basically stable. The periodic term of multiple harmonics can be used as factors, namely:

25 $${f_T}=\mathop \sum \limits_{{i=1}}^{{{m_2}}} \left[ {{b_{1i}}\sin \frac{{2\pi it}}{{365}}+{b_{2i}}\cos \frac{{2\pi it}}{{365}}} \right]$$

where *t* is the cumulative number of days from the monitoring day to the modeling start day; ${m_2}$ is the number of periodic items; ${b_{1i}}$and ${b_{2i}}$ are the regression coefficients.

Sudden changes of temperature occur occasionally in cold regions, and there is a certain deviation in using the periodic term of harmonic factor to express the temperature component. Therefore, based on the periodic term of harmonic factor and considering the impact of cold wave conditions on temperature component during dam operation, this paper constructs a sample sequence of temperature gradient, and determines cold wave conditions based on maximum entropy principle.

Assuming that the measured temperature value at time *i* in the concrete dam site area of a cold region is ${T_i}$, there are $n+1$ sets of temperature data $\left\{ {{T_0},{T_2}, \cdots {T_i}, \cdots {T_n}} \right\}$ during a certain period, and the sample space of the temperature gradient is:

26 $$y=\left\{ {{{\dot {T}}_1},{{\dot {T}}_2}, \cdots {{\dot {T}}_i}, \cdots {{\dot {T}}_n}} \right\}$$

where $${\dot {T}_i}=\frac{{{T_i} - {T_{i - 1}}}}{{{t_i} - {t_{i - 1}}}}$$.

Correspondingly, the characteristic values of the temperature gradient are represented as:

27 $${\mu _y}=\frac{1}{n}\sum {{{\left( {{{\dot {T}}_i}} \right)}^2},{\sigma _y}=\sqrt {\frac{1}{n}\left( {\sum {{{\left( {{{\dot {T}}_i}} \right)}^2} - n\mu _{y}^{2}} } \right)} }$$

where ${\mu _y}$ and ${\sigma _y}$ represent the mean and standard deviation of the sample sequence, respectively.

Considering that the temperature change during the cold wave period is a small probability event compared to the whole year, the small probability method is used to determine the boundary value of the temperature change gradient at a certain significance level $\alpha$, that is:

28 $${y_m}={F^{ - 1}}\left( {{\mu _y},{\sigma _y},\alpha } \right)$$

Based directly on numerical features, construct the maximum entropy probability density function $f(y)$. The probability distribution of the minimum deviation of sample *y* is the maximum value distribution of entropy $H(y)$ obtained based on sample information under certain constraints, that is:

29 $$\hbox{max} H\left( y \right)= - \int\limits_{R} {f\left( y \right)\ln \left( y \right)} dy$$

30 $$s.t.\int\limits_{R} {{y^i}f\left( y \right)} dy={\mu _i},i=1,2, \cdots ,n$$

where $H\left( y \right)$ is the information entropy of *y*; *R* is the integral space; ${\mu _i}$ is the *i*-th origin moment of variable *y*, ${\mu _i}=\frac{1}{n}\sum\limits_{{i=1}}^{n} {y_{i}^{2}}$.

By continuously adjusting $f\left( y \right)$ to reach the maximum entropy value $H\left( y \right)$, the Lagrange multiplier method is used to establish the Lagrange function as follows:

31 $$L=H\left( y \right)+\left( {{\lambda _0}+1} \right)\left[ {\int\limits_{R} {f\left( y \right)} dy - 1} \right]+\sum\limits_{{i=1}}^{n} {{\lambda _i}} \left[ {\int\limits_{R} {{y^i}f\left( y \right)} dy - {\mu _i}} \right]$$

where ${\lambda _i}$ is the Lagrange multiplier.

Assuming $\frac{{\partial L}}{{\partial f\left( y \right)}}=0$, then there is:

32 $$- \int\limits_{R} {\left[ {\ln f\left( y \right)+1} \right]} dy+\left( {{\lambda _0}+1} \right)\int\limits_{R} {dy} +\sum\limits_{{i=1}}^{n} {{\lambda _i}} \int\limits_{R} {{y^i}} dy=0$$

The analytical formula for further obtaining the maximum entropy probability density function $f\left( y \right)$ is

33 $$f\left( y \right)=\exp \left( {{\lambda _0}+\sum\limits_{{i=1}}^{n} {{\lambda _i}} {y^i}} \right)$$

There is an unknown Lagrange multiplier ${\lambda _i}$ in Eq. (33), which can be transformed into solving $n+1$ nonlinear equations:

34 $${G_i}\left( \lambda \right)=\int\limits_{R} {{y^i}\exp \left( {{\lambda _0}+\sum\limits_{{i=1}}^{n} {{\lambda _i}} {y^i}} \right)dy={\mu _i}}$$

The nonlinear equation system is difficult to solve using precise analytical methods and needs to be solved through Newton’s iterative method. The integration process of the above equation is solved using numerical integration method. The truncation method is used to handle the integration domain of the probability density function $f\left( y \right)$, that is, the integration values in the finite domain $\left[ {a,b} \right]$ are approximated instead of the integration values in the nonlinear domain$\left[ { - \infty ,+\infty } \right]$. The finite domain $\left[ {a,b} \right]$ is taken as $\left[ {\mu - 5\sigma ,\mu +5\sigma } \right]$, and the error of the approximation process meets the requirements of engineering accuracy.

Using the above method, the maximum entropy probability density function $f\left( y \right)$ of the random variable *y* is obtained. ${\dot {T}_m}$ is set as the critical index of temperature change gradient. Based on the small probability method, when the temperature change gradient $\dot {T}>{\dot {T}_m}$ and it is winter at that time, it is determined that the time period is a cold wave condition, and the probability is:

35 $$P\left( {\dot {T}>{{\dot {T}}_m}} \right)={P_\alpha }=\int_{{{{\dot {T}}_m}}}^{\infty } {f\left( {\dot {T}} \right)d\dot {T}} =\alpha$$

In summary, considering the winter cold wave condition as a low probability event, combined with the measured temperature data in the dam site area, the probability $\alpha$ ($\alpha$= 1%~5%) of the occurrence of the low probability event is determined. Based on this, the critical temperature gradient index ${\dot {T}_m}$ corresponding to the cold wave condition is calculated using Eq. (35), namely:

36 $${\dot {T}_m}={F^{ - 1}}\left( {\dot {T},\alpha } \right)$$

During the cold wave condition period, the temperature change gradient factor is added to the monitoring data characterization model, namely:

37 $${f_{\dot {T}}}=H\left( {{{\dot {T}}_m} - {{\dot {T}}_a}} \right)\left[ {{b_3}\Delta {T_a}+{b_4}{{\dot {T}}_a}} \right]$$

38 $$\Delta {T_a}={T_{i+1}} - {T_i};\dot {T}=\frac{{{T_{i+1}} - {T_i}}}{{{t_{i+1}} - {t_i}}}$$

where ${b_3}$ and ${b_4}$ are the coefficients; ${\dot {T}_m}$ is the limit value of temperature change gradient under cold wave conditions; ${t_i}$ refers to the accumulated days from the initial monitoring day to the cold wave period; $H\left( x \right)$ is the Heaviside step function, namely:

39 $$H(x)=\left\{ {\begin{array}{*{20}{c}} 0&{x<0} \\ 1&{x>0} \end{array}} \right.$$

Therefore, the expression of temperature component of structural state monitoring data of concrete dams in cold regions is:

40 $${f_T}=\mathop \sum \limits_{{i=1}}^{{{m_2}}} \left[ {{b_{1i}}\sin \frac{{2\pi it}}{{365}}+{b_{2i}}\cos \frac{{2\pi it}}{{365}}} \right]{\mathrm{+}}H\left( {{{\dot {T}}_m} - {{\dot {T}}_a}} \right)\left[ {{b_3}\Delta {T_a}+{b_4}{{\dot {T}}_a}} \right]$$

### Aging component ${f_\theta }$

Aging component comprehensively reflects the irreversible changes of dam concrete creep and bedrock creep. The aging component changes sharply in the initial stage of concrete dam storage, and gradually becomes stable in the later stage of operation. For concrete dams in normal operation, the linear combination of logarithm and linear function is selected to represent the aging component, namely:

41 $${f_\theta }={c_1}\theta +{c_2}\ln \theta$$

where $\theta$ is the cumulative days from the monitoring day to the initial monitoring day divided by 100; ${c_1}$ and ${c_2}$ are the regression coefficients of the aging factor.

### Freeze-thaw component ${f_I}$

Due to the special climatic conditions in the dam site, most concrete dams in cold regions have freeze-thaw conditions. The hydraulic concrete has high water saturation, and the pore water in the structure freezes and expands when the temperature drops below zero, and the ice crystals expand when the temperature rises below zero. Freeze-thaw will cause the deterioration of the concrete performance of the dam body, which will lead to changes in the monitoring data that characterizes the structural state of the dam.

Studies have shown that the changes of monitoring data caused by freeze-thaw action of dam concrete in cold regions have periodicity and hysteresis. In order to reflect the above characteristics, Eq. (42) is used to express the periodicity and hysteresis of freeze-thaw effect, and the corresponding expression of freeze-thaw component ${f_I}$ is:

42 $${f_I}=\sum\limits_{{i=2,4, \cdots }} {\left[ {{d_{i1}}\sin \frac{{2\pi i(t^{\prime} - {{t^{\prime}}_0})}}{{365}}+{d_{i2}}\cos \frac{{2\pi i(t^{\prime} - {{t^{\prime}}_0})}}{{365}}} \right]} +{d_3}{I_{i - {j_1}}}+{d_4}{I_{i - {j_2}}}+{d_5}{I_{i - {j_3}}}$$

where $t^{\prime}$ is the cumulative number of days from the monitoring day to the modeling start day; ${t^{\prime}_0}$ is the number of days from the monitoring day to the beginning day with negative temperature in the same year; ${d_{i1}},{d_{i2}},{d_3},{d_4}$ and ${d_5}$ are coefficients; $j(j={j_1},{j_2},{j_3})$ is the temperature lag term, which is the number of days calculated according to the average temperature before the *i* period and the lag period.

The ambient temperature in the dam site presents an annual periodic variation law. When the temperature of the concrete inside the dam body is lower than 0 ℃, the impact of freeze-thaw should be considered. When the temperature of the concrete in the dam body is higher than 0 ℃, the impact of freeze-thaw should not be considered. Therefore, the Heaviside step function is added, namely:

43 $$H(T)=\left\{ {\begin{array}{*{20}{c}} 0&{T>0} \\ 1&{T<0} \end{array}} \right.$$

For the concrete temperature *T*, the actual measured value of the thermometer inside the dam body or the temperature boundary data such as air temperature and water temperature are used to calculate the temperature field distribution of the dam body. The negative temperature area and negative temperature period of the concrete dam are judged according to the position of the monitoring point. In the area of ​​the negative temperature period, add the freeze-thaw factor.

Substituting Eq. (43) into Eq. (42), the expression of the freeze-thaw component of structural state monitoring data of concrete dams in cold regions is obtained as:

44 $${f_I}=H(T) \cdot \left[ {\sum\limits_{{i=2,4}}^{{{m_4}}} {\left( {{d_{i1}}\sin \frac{{2\pi i(t^{\prime} - {{t^{\prime}}_0})}}{{365}}+{d_{i2}}\cos \frac{{2\pi i(t^{\prime} - {{t^{\prime}}_0})}}{{365}}} \right)+} {d_3}{I_{i - {j_1}}}+{d_4}{I_{i - {j_2}}}+{d_5}{I_{i - {j_3}}}} \right]$$

### Component of overwintering layer ${f_Y}$

**Fig. 3:**
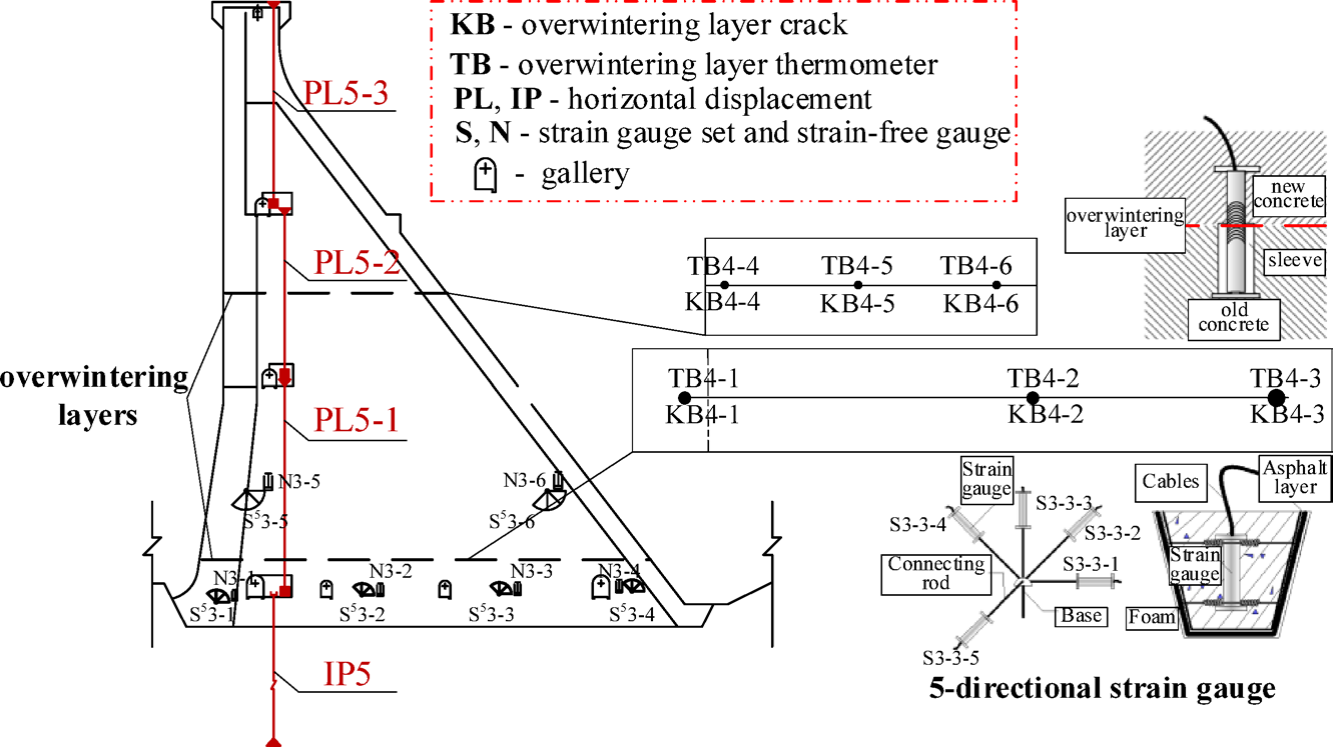
Distribution map of strain gauges and joint gauges near the wintering layer of a certain dam.

The winter layers in concrete dams in cold regions may affect the structural properties of concrete dams. Joint gauges are primarily embedded in the tensile zone of the dam body, the junction of the dam body and the bank slope, areas with sudden changes in bedrock elevation, and the construction wintering layer. Strain gauges are primarily embedded upstream and downstream of different dam sections, as well as in the overwintering layer. To monitor the properties of the overwintering layer, joint gauges were specifically embedded in the overwintering layer to describe its changes and enable timely action when abnormal changes are detected. Therefore, this section characterizes the influence of the overwintering layer based on the data of the strain gages and seam gauges near the overwintering layers. Figure 3 shows the distribution of strain gauges and joint gauges near the wintering layer of a certain dam.

The wintering layer component expression is:

45 $${f_Y}=\sum\limits_{{i=1}}^{{{m_5}}} {{e_{1i}}{\varepsilon _i}+} \sum\limits_{{j=1}}^{{{m_6}}} {{e_{2j}}{J_j}}$$

where ${e_{1i}}$ and ${e_{2j}}$ are the coefficients; ${\varepsilon _i}$ and ${J_j}$ are the measured values of the $i - th$ strain gauge and the $j - th$ branch near the wintering layer, respectively.

The construction principles of the influence factors of the monitoring variables such as stress and strain, are similar to the above-mentioned deformation, and will not be repeated here.

## Characterization model of the structural performance monitoring data of concrete dams in cold regions

A large number of monitoring instruments are embedded in different positions of the concrete dam to comprehensively reflect the change characteristics of its structural change characteristics. Based on the measured data, the construction method of the monitoring data characterization model of the structural performance of concrete dams in cold regions is explored. In view of the characteristics of structural state changes of concrete dams in cold regions and the correlation between the monitoring data of various measuring points, this section introduces spatial coordinate variables and establishes a multi-measuring point monitoring data characterization model to understand the overall spatial distribution law of monitoring data under the load combination at a certain time.

In the spatial coordinate system, the monitoring data of the concrete dam structural state is expressed as:

46 $$\begin{aligned} f\left( {x,y,z} \right) & = f_{1} \left( {H,x,y,z} \right) + f_{2} \left( {T,x,y,z} \right) + f_{3} \left( {\theta ,x,y,z} \right) + f_{4} \left( {I,x,y,z} \right) + f_{5} \left( {Y,x,y,z} \right) \\ & = f_{1} \left[ {f\left( H \right),g\left( {x,y,z} \right)} \right] + f_{2} \left[ {f\left( T \right),g\left( {x,y,z} \right)} \right] + f_{3} \left[ {f\left( \theta \right),g\left( {x,y,z} \right)} \right] \\ & + f_{4} \left[ {f\left( I \right),g\left( {x,y,z} \right)} \right] + f_{5} \left[ {f\left( Y \right),g\left( {x,y,z} \right)} \right] \\ \end{aligned}$$

where ${f_1}\left( {H,x,y,z} \right)$,${f_2}\left( {T,x,y,z} \right)$,${f_3}\left( {\theta ,x,y,z} \right)$,${f_4}\left( {I,x,y,z} \right)$ and ${f_5}\left( {Y,x,y,z} \right)$ are the water pressure component, temperature component, aging component, freeze-thaw component and wintering layer component of multiple measuring points, respectively; $f\left( H \right),f\left( T \right),f\left( \theta \right),f\left( I \right)$ and $f\left( Y \right)$ are the water pressure component, temperature component, aging component, freeze-thaw component and wintering layer component, constructed in Sect. 3; $g\left( {x,y,z} \right)$ is the characteristic function of spatial distribution.

### Water pressure component of multiple measuring points ${f_1}\left( {H,x,y,z} \right)$

Water pressure component of multiple measuring points is comprehensively represented by the water pressure component in Sect. 3 and the spatial distribution characteristic function, namely:

47 $${f_1}\left( {H,x,y,z} \right){\text{= }}{f_1}\left[ {f\left( H \right),g\left( {x,y,z} \right)} \right]$$

where $f\left( H \right)$is the water pressure component, expressed by Eq. (21); $g\left( {x,y,z} \right)$ is continuous in the definition domain, expanded by multivariate power series, and obtained by taking the trinomial formula:

48 $$g\left( {x,y,z} \right){\mathrm{=}}\sum\limits_{{l,m,n=0}}^{3} {{a_{lmn}}{x^l}{y^m}{z^n}}$$

Equation (47) is converted into:

49 $${f_1}\left( {H,x,y,z} \right){\text{ = }}{f_1}\left[ {\sum\limits_{{i=0}}^{{3(4)}} {{a_i}{H^i}} ,\sum\limits_{{l,m,n=0}}^{3} {{a_{lmn}}{x^l}{y^m}{z^n}} } \right]$$

Similarly, the multivariate power series expansion is adopted and the same terms are merged. The expression of the water pressure component of multiple monitoring points of the concrete dam is as follows:

50 $${f_1}\left( {H,x,y,z} \right){\mathrm{=}}\sum\limits_{{i=0}}^{{3(4)}} {\sum\limits_{{l,m,n=0}}^{3} {{A_{klmn}}{H^k}{x^l}{y^m}{z^n}} }$$

where ${A_{klmn}}$ is the coefficient.

### Temperature component of multiple measuring points ${f_2}\left( {T,x,y,z} \right)$

Similarly, under the action of temperature change, the temperature component of multiple measuring points of concrete dam structural state is comprehensively expressed by the temperature component and the spatial distribution characteristic function, and its expression is:

51 $${f_2}\left( {T,x,y,z} \right)={f_2}\left[ {f\left( T \right),g\left( {x,y,z} \right)} \right]$$

where: $f\left( T \right)$ refers to the temperature component, expressed by Eq. (22), (25), added with Eq. (37).

The expression of temperature component of multiple measuring point monitoring expressed by equivalent temperature is:

52 $$\begin{aligned} f_{2} \left( {T,x,y,z} \right) & = \sum\limits_{{j,k = 1}}^{{m_{2} }} {\sum\limits_{{l,m,n = 0}}^{3} {B_{{jklmn}} \bar{T}^{j} \beta ^{k} x^{l} y^{m} z^{n} } } \\ & + H\left( {\dot{T}_{m} - \dot{T}_{a} } \right) \cdot \sum\limits_{{j = 0}}^{1} {\sum\limits_{{l,m,n = 0}}^{3} {B_{{jlmn}} \Delta T_{a}^{j} \dot{T}_{a}^{{1 - j}} x^{l} y^{m} z^{n} } } \\ \end{aligned}$$

The expression of temperature component of multiple measuring point monitoring expressed by harmonic function factor is:

53 $$\begin{aligned} f_{2} \left( {T,x,y,z} \right) & = \sum\limits_{{j,k = 0}}^{1} {\sum\limits_{{l,m,n = 0}}^{3} {B_{{jklmn}} \sin \frac{{2\pi jt}}{{365}}\cos \frac{{2\pi kt}}{{365}}x^{l} y^{m} z^{n} } } \\ & + H\left( {\dot{T}_{m} - \dot{T}_{a} } \right) \cdot \sum\limits_{{j = 0}}^{1} {\sum\limits_{{l,m,n = 0}}^{3} {B_{{jlmn}} \Delta T_{a}^{j} \dot{T}_{a}^{{1 - j}} x^{l} y^{m} z^{n} } } \\ \end{aligned}$$

${B_{jklmn}}$ and ${B_{jlmn}}$ are various coefficients.

### Aging component of multiple measuring points ${f_3}\left( {\theta ,x,y,z} \right)$

Aging component of multiple measuring points is expressed as:

54 $${f_3}\left( {\theta ,x,y,z} \right)={f_3}\left[ {f\left( \theta \right),g\left( {x,y,z} \right)} \right]$$

where $f\left( \theta \right)$ is the aging component, which is expressed as Eq. (41).

The specific aging component of multiple measuring points expression of Eq. (54) is:

55 $${f_3}\left( {\theta ,x,y,z} \right)=\sum\limits_{{j,k=0}}^{1} {\sum\limits_{{l,m,n=0}}^{3} {{C_{jklmn}}{\theta _j}\ln {\theta _k}{x^l}{y^m}{z^n}} }$$

where ${C_{jklmn}}$ is the coefficient.

### Freeze-thaw component of multiple measuring points${f_4}\left( {I,x,y,z} \right)$

For the actual working conditions in cold regions, the expression form of freeze-thaw component is proposed in Sect. 3 of this paper. Based on this, the expression of freezing and thawing components measured at multiple measuring points is as follows:

56 $${f_4}\left( {I,x,y,z} \right)={f_4}\left[ {f\left( I \right),g\left( {x,y,z} \right)} \right]$$

where $f\left( I \right)$ is the freeze-thaw component, expressed by Eq. (44).

The specific expression of freeze-thaw component of multiple measuring points in corresponding to Eq. (56) is:

57 $${f_4}\left( {I,x,y,z} \right)=H(T) \cdot \left[ \begin{gathered} \sum\limits_{{j,k=0}}^{2} {\sum\limits_{{l,m,n=0}}^{3} {\left( {{D_{jklmn}}\sin \frac{{2\pi j(t^{\prime} - {{t^{\prime}}_0})}}{{365}}\cos \frac{{2\pi j(t^{\prime} - {{t^{\prime}}_0})}}{{365}}{x^l}{y^m}{z^n}} \right)} } \hfill \\ +\sum\limits_{{j=1,2,4}}^{4} {\sum\limits_{{l,m,n=0}}^{3} {\left( {{D_{jlmn}}{I_{i - {j_1}}}{x^l}{y^m}{z^n}} \right)} } \hfill \\ \end{gathered} \right]$$

where ${D_{jklmn}}$ and ${D_{jlmn}}$ are various coefficients.

### Overwintering layer component of multiple measuring points ${f_5}\left( {Y,x,y,z} \right)$

In Sect. 3, a method is proposed to characterize the overwintering layer component by using the data of strain gauges and joint gauges near the overwintering layer. The expression of overwintering layer component of multiple measuring points can be expressed as:

58 $${f_5}\left( {Y,x,y,z} \right)={f_5}\left[ {f\left( Y \right),g\left( {x,y,z} \right)} \right]$$

The specific expression of overwintering layer component of multiple measuring points in corresponding to Eq. (58) is:

59 $${f_5}\left( {Y,x,y,z} \right)=\sum\limits_{{i=1}}^{{{m_5}}} {\sum\limits_{{l,m,n=0}}^{3} {{E_{1ilmn}}{\varepsilon _i}{x^l}{y^m}{z^n}} +} \sum\limits_{{j=1}}^{{{m_6}}} {{E_{2jlmn}}{J_j}{x^l}{y^m}{z^n}}$$

where ${E_{1ilmn}}$ and ${E_{2jlmn}}$are various coefficients; The meanings of ${\varepsilon _i}$ and ${J_j}$ are the same as Eq. (45).

When there is continuous thermometer data in the dam body, the expression of characterization model of the multiple measuring points is as follows using the equivalent temperature method:

60 $$\begin{aligned} f\left( {x,y,z} \right) & = \sum\limits_{{i = 0}}^{{3(4)}} {\sum\limits_{{l,m,n = 0}}^{3} {A_{{klmn}} H^{k} x^{l} y^{m} z^{n} } } \\ & + \sum\limits_{{j,k = 1}}^{{m_{2} }} {\sum\limits_{{l,m,n = 0}}^{3} {B_{{jklmn}} \bar{T}^{j} \beta ^{k} x^{l} y^{m} z^{n} } } \\ & + H\left( {\dot{T}_{m} - \dot{T}_{a} } \right) \cdot \sum\limits_{{j = 0}}^{1} {\sum\limits_{{l,m,n = 0}}^{3} {B_{{jlmn}} \Delta T_{a}^{j} \dot{T}_{a}^{{1 - j}} x^{l} y^{m} z^{n} } } \\ & + \sum\limits_{{j,k = 0}}^{1} {\sum\limits_{{l,m,n = 0}}^{3} {C_{{jklmn}} \theta _{j} \ln \theta _{k} x^{l} y^{m} z^{n} } } \\ & + H(T) \cdot \left[ \begin{gathered} \sum\limits_{{j,k = 0}}^{2} {\sum\limits_{{l,m,n = 0}}^{3} {\left( {D_{{jklmn}} \sin \frac{{2\pi j(t^{\prime} - t^{\prime}_{0} )}}{{365}}\cos \frac{{2\pi j(t^{\prime} - t^{\prime}_{0} )}}{{365}}x^{l} y^{m} z^{n} } \right)} } \hfill \\ + \sum\limits_{{j = 1,2,4}}^{4} {\sum\limits_{{l,m,n = 0}}^{3} {\left( {D_{{jlmn}} I_{{i - j_{1} }} x^{l} y^{m} z^{n} } \right)} } \hfill \\ \end{gathered} \right] \\ & + \sum\limits_{{i = 1}}^{{m_{5} }} {\sum\limits_{{l,m,n = 0}}^{3} {E_{{1ilmn}} \varepsilon _{i} x^{l} y^{m} z^{n} } + } \sum\limits_{{i = 1}}^{{m_{6} }} {E_{{2jlmn}} J_{i} x^{l} y^{m} z^{n} } \\ \end{aligned}$$

When multiple groups of harmonic factors are used to describe the temperature component, the expression of measurement characterization model of the multiple measuring points for the structural state of concrete dams in cold regions is as follows:

61 $$\begin{aligned} f\left( {x,y,z} \right) & = \sum\limits_{{i = 0}}^{{3(4)}} {\sum\limits_{{l,m,n = 0}}^{3} {A_{{klmn}} H^{k} x^{l} y^{m} z^{n} } } \\ & + \sum\limits_{{j,k = 0}}^{1} {\sum\limits_{{l,m,n = 0}}^{3} {B_{{jklmn}} \sin \frac{{2\pi jt}}{{365}}\cos \frac{{2\pi kt}}{{365}}x^{l} y^{m} z^{n} } } \\ & + H\left( {\dot{T}_{m} - \dot{T}_{a} } \right) \cdot \sum\limits_{{j = 0}}^{1} {\sum\limits_{{l,m,n = 0}}^{3} {B_{{jlmn}} \Delta T_{a}^{j} \dot{T}_{a}^{{1 - j}} x^{l} y^{m} z^{n} } } \\ & + \sum\limits_{{j,k = 0}}^{1} {\sum\limits_{{l,m,n = 0}}^{3} {C_{{jklmn}} \theta _{j} \ln \theta _{k} x^{l} y^{m} z^{n} } } \\ & + H(T) \cdot \left[ \begin{gathered} \sum\limits_{{j,k = 0}}^{2} {\sum\limits_{{l,m,n = 0}}^{3} {\left( {D_{{jklmn}} \sin \frac{{2\pi j(t^{\prime} - t^{\prime}_{0} )}}{{365}}\cos \frac{{2\pi j(t^{\prime} - t^{\prime}_{0} )}}{{365}}x^{l} y^{m} z^{n} } \right)} } \hfill \\ + \sum\limits_{{j = 1,2,4}}^{4} {\sum\limits_{{l,m,n = 0}}^{3} {\left( {D_{{jlmn}} I_{{i - j_{1} }} x^{l} y^{m} z^{n} } \right)} } \hfill \\ \end{gathered} \right] \\ & + \sum\limits_{{i = 1}}^{{m_{5} }} {\sum\limits_{{l,m,n = 0}}^{3} {E_{{1ilmn}} \varepsilon _{i} x^{l} y^{m} z^{n} } + } \sum\limits_{{i = 1}}^{{m_{6} }} {E_{{2jlmn}} J_{i} x^{l} y^{m} z^{n} } \\ \end{aligned}$$

In order to reflect the local state change of the dam at the specific measuring point, the above equation is degraded for a single measuring point, and the single measuring point characterization model is obtained as follows:

62 $$\begin{aligned} f & = f_{H} + f_{T} + f_{\theta } + f_{I} + f_{Y} \\ & = a_{0} + \mathop \sum \limits_{{i = 1}}^{{m_{1} }} a_{i} H^{i} + \mathop \sum \limits_{{i = 1}}^{{m_{2} }} \left[ {b_{{1i}} \sin \frac{{2\pi it}}{{365}} + b_{{2i}} \cos \frac{{2\pi it}}{{365}}} \right] \\ & + H\left( {\dot{T}_{m} - \dot{T}_{a} } \right)\left[ {b_{3} \Delta T_{a} + b_{4} \dot{T}_{a} } \right] + c_{1} \theta + c_{2} \ln \theta \\ & + H(T) \cdot \left[ {\sum\limits_{{i = 2,4, \cdots }}^{{m_{4} }} {\left( {d_{{i1}} \sin \frac{{2\pi i(t^{\prime} - t^{\prime}_{0} )}}{{365}} + d_{{i2}} \cos \frac{{2\pi i(t^{\prime} - t^{\prime}_{0} )}}{{365}}} \right)} + d_{3} I_{{i - j_{1} }} + d_{4} I_{{i - j_{2} }} + d_{5} I_{{i - j_{3} }} } \right] \\ & + \sum\limits_{{i = 1}}^{{m_{5} }} {e_{{1i}} \varepsilon _{i} + } \sum\limits_{{j = 1}}^{{m_{6} }} {e_{{2j}} J_{i} } \\ \end{aligned}$$

The meanings of all the parameters in the equations are the same as before.

**Fig. 4:**
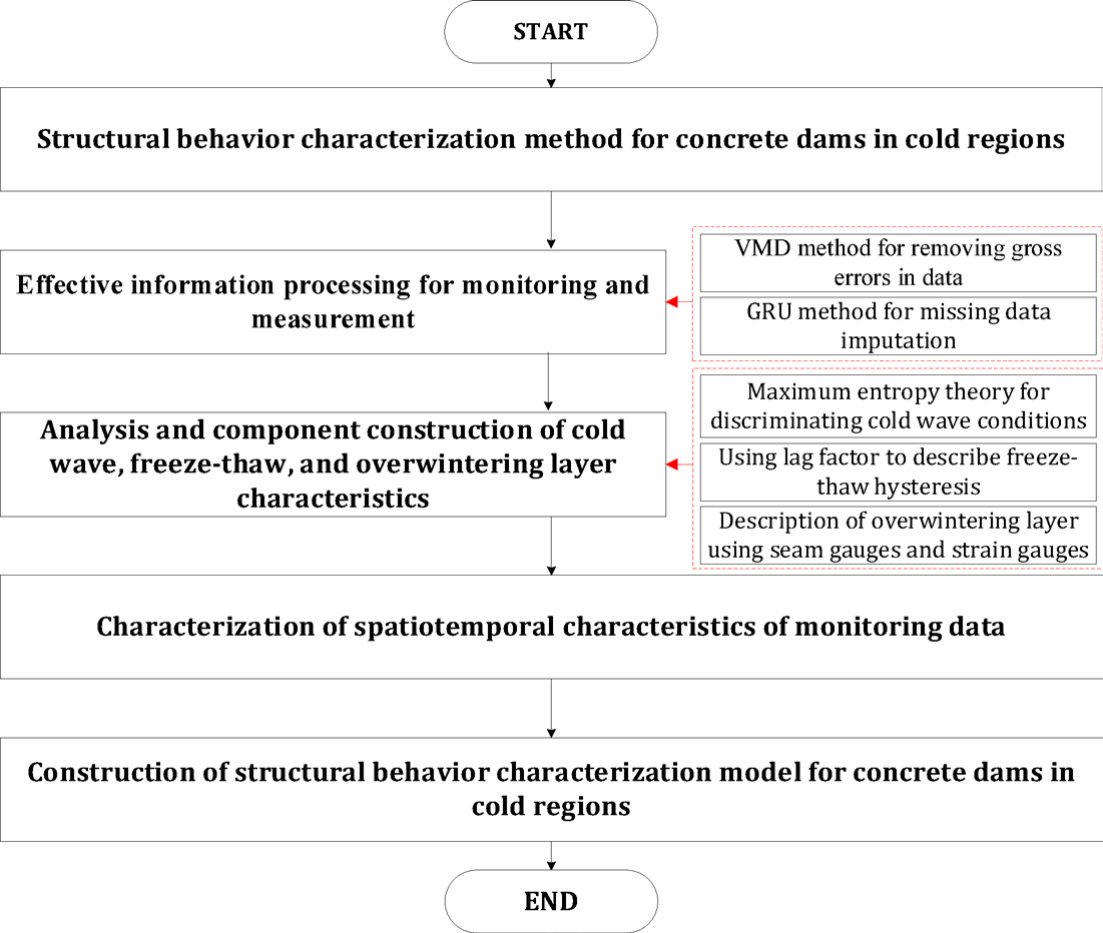
Schematic diagram of the model architecture.

Therefore, the schematic diagram of the model architecture in this manuscript is shown in Fig. 4.

## Engineering project

**Fig. 5:**
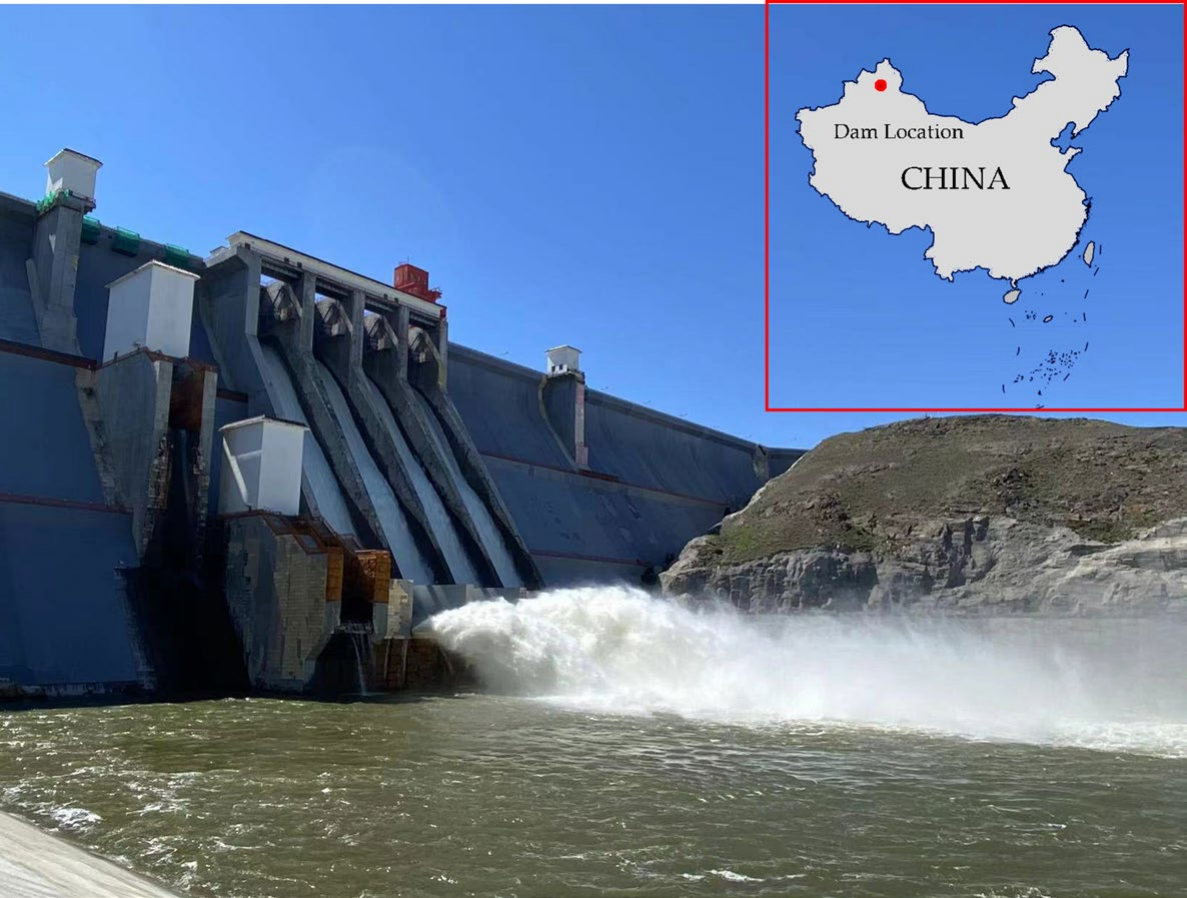
A concrete gravity dam in a cold region (This image is created by the author using Microsoft Office PowerPoint and the URL is https://www.microsoftstore.com.cn/software/office/powerpoint-2024).

**Fig. 6:**
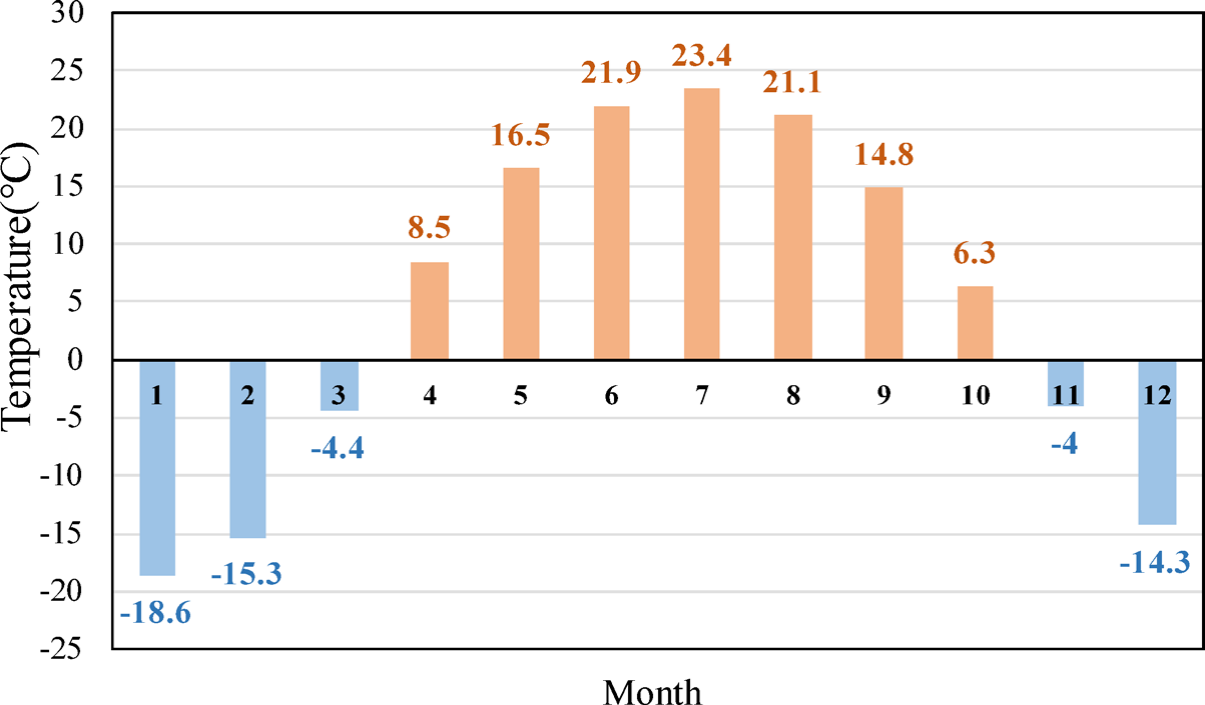
Monthly average temperature statistics of the dam site over the years Unit:℃.

A concrete gravity dam with a maximum height of 121.5 m is located in a high latitude region of China, where winters are extremely cold and long. The engineering grade is Class 1, with a design flood return period of 1000 years (*P* = 0.1%) and a check flood return period of 5000 years (*P* = 0.02%). Figure 5 shows the real picture and the location of the dam. The coldest month is January, with the lowest temperature reaching − 18.6 °C and the annual average temperature over the years 4.7 °C. The average temperature of each month in the dam site is shown in Fig. 6.

**Fig. 7:**
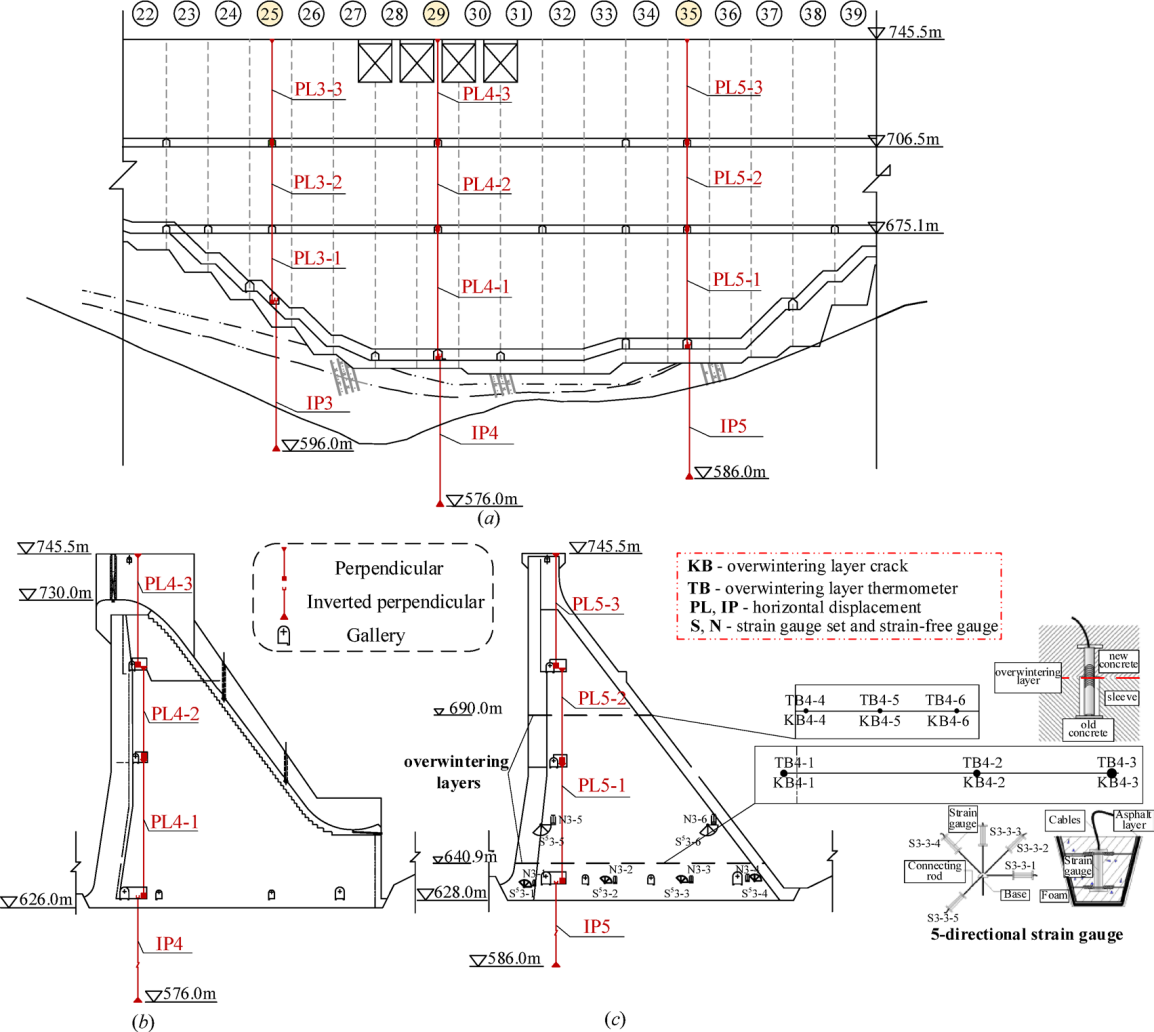
Typical pendulums and instruments layout of part of the riverbed of a concrete dam in cold regions.

The dam is observed by a group of pendulums, which are set in a typical dam section with complex geology or structure. Typical pendulums and some instruments layout of part of the dam riverbed is shown in Fig. 7.

A large number of measuring and monitoring instruments are buried the construction of the project. Here is a brief introduction to the main instruments. Strain gauges, joint gauges, and thermometers are selected from differential resistance instruments developed and produced by Chinese companies, while pendulum instruments are selected from intelligent capacitive instruments. The parameters of the relevant instruments and equipment are shown in the Table 1.

**Table 1:** The parameters of the relevant instruments and equipment.

Measuring instrument	Specification code	Operating range	Accuracy	Allowableo perating temperature
Pendulum instrument	RZ-S	± 25 mm	≤ 0.5%F.S.	−25 ~ + 60℃
5 directional strain gauge set	NZS-25G	+ 600ཞ−1000µε	≤ 3µε/0.01%	−25 ~ + 60℃
Joint gauge	NZJ-12	12 mm	≤ 0.3%F.S	−25 ~ + 60℃
Thermometers	NZWD	−30ཞ+70 ℃	± 0.3 ℃	/

### Extraction of effective monitoring information

#### Identification and processing of gross errors in monitoring data

**Fig. 8:**
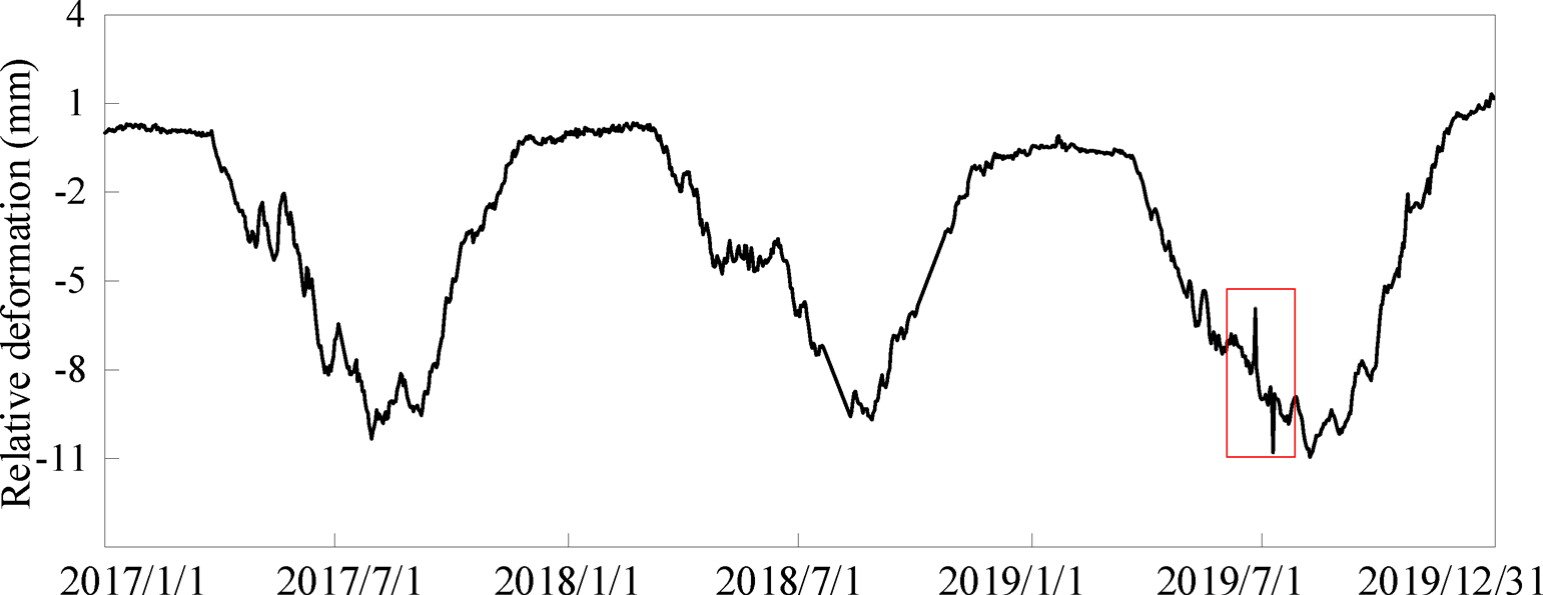
Relative deformation along the river of point PL4-3 on 29^#^ dam section crest.

The measured data of typical measuring point PL4-3 of 29^#^ dam section along the river (compared with the relative deformation of the starting day of the sequence) are selected. The time series is from January 1, 2017 to December 31, 2019, with a total of 1015 monitoring values. Two gross errors are artificially added to the monitoring data sequence to verify the effectiveness of identification and processing methods for gross errors in Sect. 2. The artificially added gross errors are marked or bolded in Fig. 8; Table 2.

**Table 2:** Local sequence of measured data of deformation with gross error added at measuring point PL4-3 (mm).(The two bolded values are manually added gross errors)

Date	Original monitoring sequence	Sequence with gross errors
…	…	…
2019/6/23	−8.09	−8.09
2019/6/24	−7.77	−7.77
2019/6/25	−7.69	−7.69
2019/6/26	−7.53	**−5.93**
2019/6/27	−7.96	−7.96
2019/6/28	−8.31	−8.31
2019/6/29	−8.68	−8.68
2019/6/30	−8.94	−8.94
2019/7/1	−9.00	−9.00
2019/7/2	−8.98	−8.98
2019/7/3	−8.97	−8.97
2019/7/4	−8.83	−8.83
2019/7/5	−9.06	−9.06
2019/7/6	−9.19	−9.19
2019/7/7	−8.75	**−10.4**
2019/7/8	−8.58	−8.58
2019/7/9	−9.14	−9.14
…	…	…

**Fig. 9:**
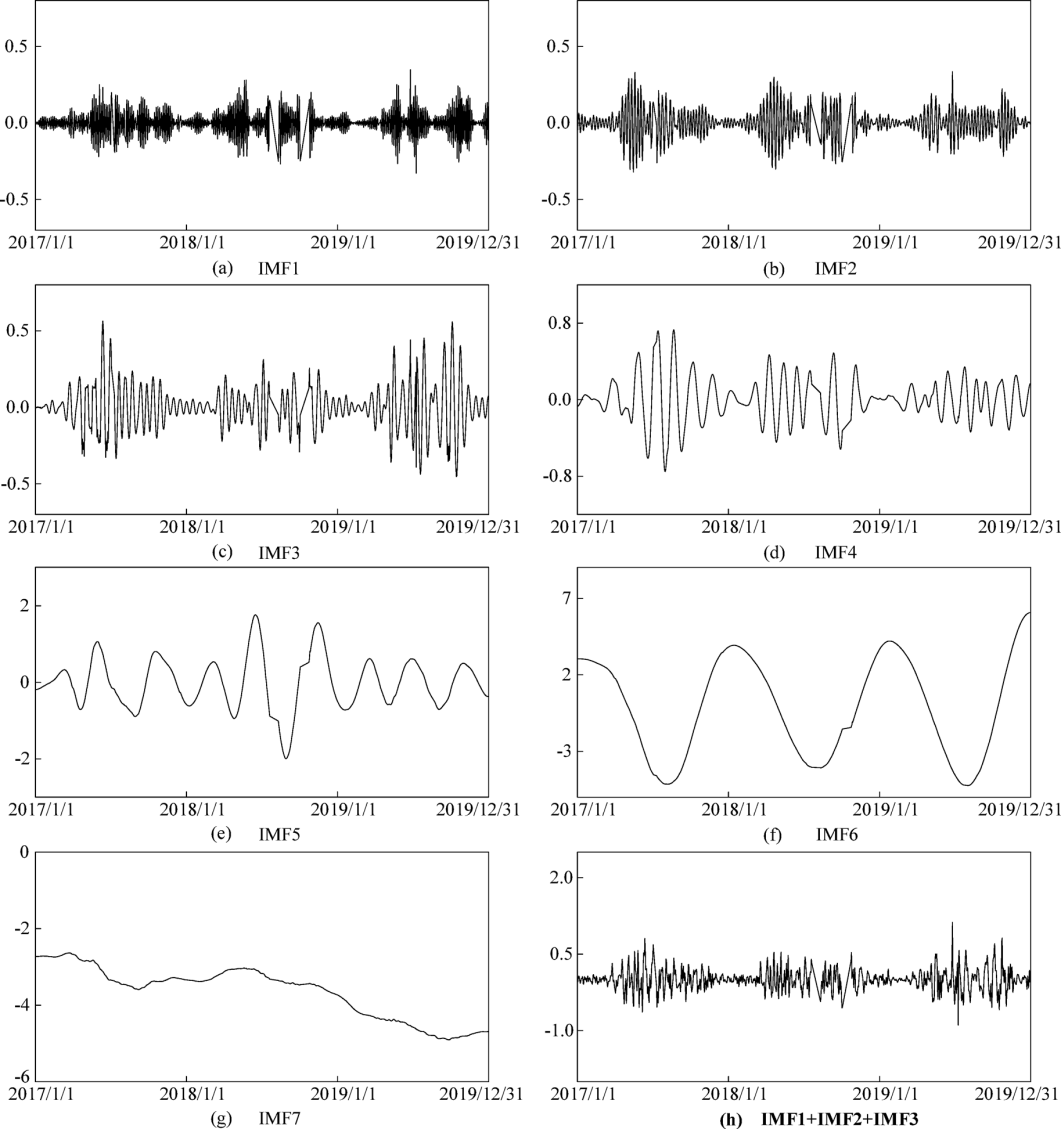
Variational modal decomposition results of relative deformation along the river of measuring point PL4-3 (mm).

Firstly, the above monitoring data with gross errors are decomposed by VMD method. Referring to other relevant literature, the value of K is usually between 4 and 10. By using the trial and error method on the current data sequence, the K value is determined to be 7. Figure 9(a, g) are the modal components obtained by decomposition. It can be seen that IMF1 ~ 3 are high -frequency components, IMF4 ~ 7 are low-frequency components, and the stacked IMF1 ~ 3 is used as high-frequency component sequence for gross error processing.

According to the high-frequency component sequence separated by the VMD method, the initial standard deviation ${\sigma _n}$ is 0.254, the minimum posterior standard deviation ${\sigma _{n - 1}}$ is 0.221, and the statistical inspection quantity ${\gamma _1}$ is 1.149. Therefore, ${\gamma _1}>{F_{0.05}}\left( {1015,1014} \right)=1.11$, and the first gross error of the sequence can be determined. Repeat the above steps, the second search shows that ${\sigma _{n - 2}}$is 0.198, and ${\gamma _2}$ is 1.116. Therefore, ${\gamma _2}>{F_{0.05}}\left( {1014,1013} \right)=1.11$, and the second gross error is determined further. After the third search, the minimum posterior standard deviation${\sigma _{n - 3}}$ is 0.196, ${\gamma _3}$ is 1.010 and ${\gamma _3}<{F_{0.05}}\left( {1013,1012} \right)=1.11$, which indicates that there is no significant change in the sequence standard deviation after removing the third value. The preliminary identification of gross error is over. In the above process, this paper also analyzes the environment and load changes in the corresponding period, and the results show that there is no sudden change in the corresponding load at the time of suspected gross error, which reflects that the sudden change of deformation measurement value is caused by gross error.

At the same time, using the “3S” confidence interval determined by the HST (hydrostatic-seasonal-time) statistical model, the difference between the identified gross error data and the measured data is 1.86 mm. At this time, the two preset gross error data cannot be identified. However, the gross error identification and processing method based on the VMD method can accurately identify and eliminate the preset gross error. In addition, this method does not need to establish HST statistical model in advance. Therefore, the effectiveness of the gross error identification and elimination method proposed in this paper can be verified.

#### Processing of monitoring data missing

According to the GRU neural network deep learning algorithm, the missing data processing of the measurement sequence is carried out. It is artificially assumed that the PL4-3 measuring point data is missing from October 3, 2019 to October 30, 2019 in the relative deformation along the river. The selection of GRU parameters mainly considers factors such as model complexity, data size, and computing resources, and is based on common range intervals. Batch_size refers to the number of samples processed simultaneously during model training or inference, learning_rate is usually between 0.01 and 0.1 and num_hiddens is usually between 20 and 100. Therefore, After multiple experiments, the values of the hyperparameters were adjusted to batch_size of 30, learning_rate of 0.02, and num_hiddens of 64. After 300 times of learning and training, the GRU neural network model is obtained. The trained GRU neural network model is used to impute missing values. To facilitate comparison, the HST statistical model imputation and linear imputation results are calculated respectively, as shown in Table 3.

**Table 3:** Interpolation results of continuous missing data of multiple methods unit: mm. (The bolded values represent the root mean square error of the GRU algorithm proposed in this article)

Date	Original value	GRU interpolation results	HST statistical model interpolation results	Linear interpolation results
2019/10/2	−6.31	−6.22	−6.26	−6.31
2019/10/3	−6.00	−5.99	−6.17	−6.17
2019/10/4	−5.55	−5.54	−5.86	−6.03
2019/10/5	−5.48	−5.45	−5.66	−5.90
2019/10/6	−5.32	−5.32	−5.50	−5.76
2019/10/7	−5.31	−5.31	−5.18	−5.62
2019/10/8	−6.07	−6.03	−5.13	−5.48
2019/10/9	−4.48	−4.50	−5.07	−5.35
2019/10/10	−4.66	−4.66	−4.89	−5.21
2019/10/11	−5.10	−5.08	−4.96	−5.07
2019/10/12	−5.03	−5.02	−5.06	−4.93
2019/10/13	−5.06	−5.05	−5.02	−4.80
2019/10/14	−4.63	−4.63	−4.83	−4.66
2019/10/15	−4.82	−4.82	−4.85	−4.52
2019/10/16	−4.70	−4.70	−4.88	−4.38
2019/10/17	−4.40	−4.41	−4.59	−4.25
2019/10/18	−4.37	−4.39	−4.63	−4.11
2019/10/19	−4.01	−4.02	−4.53	−3.97
Root mean square error	/	**0.012**	0.256	0.288

As can be seen from Table 3, the deep learning algorithm based on GRU neural network proposed is obviously superior to the other two algorithms in the interpolation of missing data, which can be applied to the process of missing measurement data of concrete dams. The results have verified the effectiveness of the missing data processing method proposed in this paper.

### Characterization model for monitoring sequence of concrete dam structural behavior in cold regions

Based on the above gross error identification and missing data processing methods, the effective information of the monitoring sequence is extracted. On this basis, combined with the operating characteristics of concrete dams in cold regions, and considering the effects of actual temperature change and freeze-thaw in cold regions, the expression of the characterization model of the monitoring sequence of concrete dam deformation along the river direction is established according to Eq. (61).

**Fig. 10:**
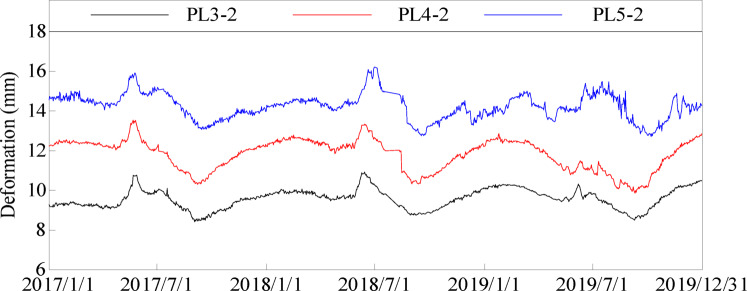
Deformation process line of typical measuring points of 25^#^,29^#^ and 35^#^ dam sections.

In this section, the measured deformation data of typical measuring points PL3-2, PL4-2 and PL5-2 at the same elevation of 25^#^, 29^#^ and 35^#^ dam sections are selected as the research object. The modeling period is from January 1, 2017 to December 31, 2019. The VMD method and GRU neural network method are used to eliminate the gross errors of each sequence and interpolate the missing data, and effective information is extracted. The deformation values of each measuring point along the river are shown in Fig. 10.

**Fig. 11:**
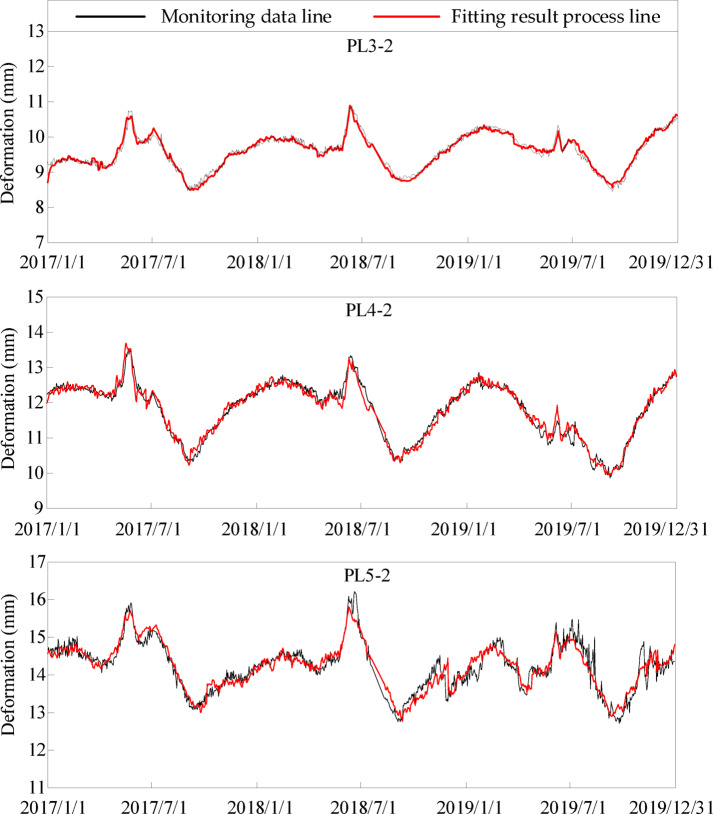
Deformation fitting process line of multiple measuring points of 25^#^, 29^#^ and 35^#^ dam sections.

Based on the deformation monitoring data information of the above measuring points, considering the joint influence of temperature change, freeze-thaw and overwintering layer on the structural behavior of concrete dams, the corresponding influencing factors’ expressions are established. The deformation characterization model of the structural behavior change of concrete dams is constructed as shown in Eq. (61). Figure 11 shows the monitoring data and fitting results of each measuring point.

It can be seen from Fig. 11 that the characterization model proposed in this paper has high accuracy and can accurately characterize the changes in structural behavior of concrete dams in cold regions.

**Fig. 12:**
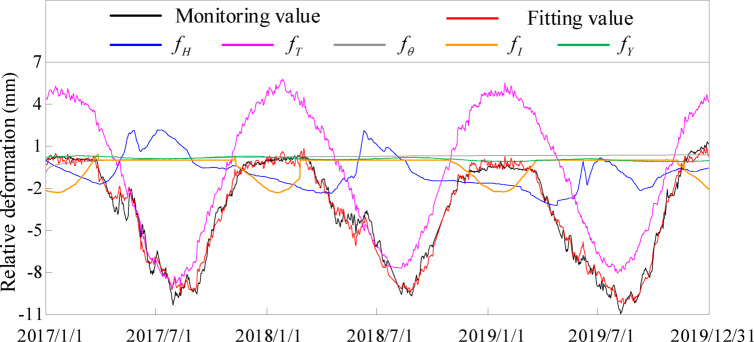
Relative deformation components along the river of point PL4-3 on 29^#^ dam section crest.

In order to reflect the influence of cold wave, freeze-thaw and overwintering layer, it is necessary to separate the characterization model components. Taking typical measuring point PL4-3 of dam Section 29^#^ in the downstream deformation direction (relative deformation compared to the starting date of the sequence) as an example, after extracting the effective monitoring information, the modeling results of the downstream deformation of dam crest of dam Section 29^#^, as well as the components and coefficients of the characterization model, are obtained by substituting it into the characterization model established by Eq. (62), as shown in Fig. 12; Table 4,  5. The positive monitoring data in the example of this article indicates downstream deformation of the dam body.

**Table 4:** Coefficients for deformation characterization of a dam in cold area.

Coefficient	Value	Coefficient	Value
${a_0}$	−1.701	${d_{21}}$	−1.155
${a_1}$	−52.106	${d_{22}}$	−1.304
${a_2}$	0.465	${d_{41}}$	−0.498
${a_3}$	−0.001	${d_{42}}$	0.852
${b_{11}}$	2.659	${d_3}$	0
${b_{12}}$	5.474	${d_4}$	−0.025
${b_{21}}$	−0.668	${d_5}$	0
${b_{22}}$	−0.246	${e_{11}}$	0
${b_3}$	−0.057	${e_{12}}$	0
${b_4}$	0.048	${e_{21}}$	0.002
${c_1}$	0	${e_{22}}$	0
${c_2}$	0.185		

**Table 5:** Comparison of fitting effect of deformation analysis model.

Model type	Multiple correlation coefficient *R*	Root mean square error S (mm)
HST statistical model	0.9640	0.6221
LSTM model	0.9879	0.4403
The model proposed in this paper	0.9898	0.4200

The model expression for the monitoring of the dam crest of the 29^#^ dam section of a concrete dam in the cold region along the river direction deformation is:

The expression of the characterization model of the deformation monitoring sequence along the river at the crest of 29^#^ dam section of a concrete dam in the cold region is as follows:

63 $$\begin{aligned} \delta & = \delta _{H} + \delta _{T} + \delta _{\theta } + \delta _{I} + \delta _{Y} \\ & = - 1.701 - 52.106\left( {H - H_{0} } \right) + 0.465\left( {H^{2} - H_{0}^{2} } \right) - 0.001\left( {H^{3} - H_{0}^{3} } \right) \\ & + 2.659\sin \frac{{2\pi t}}{{365}} + 4.574\cos \frac{{2\pi t}}{{365}} - 0.668\sin \frac{{4\pi t}}{{365}} - 0.246\cos \frac{{4\pi t}}{{365}}H\left( {\dot{T}_{m} - \dot{T}_{a} } \right) \cdot \left( { - 0.057\Delta T_{a} + 0.048\dot{T}_{a} } \right) \\ & + 0.185\ln \theta + H(T) \cdot \left[ \begin{gathered} - 1.155\sin \frac{{4\pi (t^{\prime} - 304)}}{{365}} - 1.304\cos \frac{{4\pi (t^{\prime} - 304)}}{{365}} \hfill \\ - 0.498\sin \frac{{8\pi (t^{\prime} - 304)}}{{365}} + 0.852\cos \frac{{8\pi (t^{\prime} - 304)}}{{365}} - 0.025I_{{15 - 7}} \hfill \\ \end{gathered} \right] + 0.002J_{1} \\ \end{aligned}$$

where ${I_{15 - 7}}$ represents the temperature lag factor with 15 days and 7 days calculated according to the average temperature; ${J_1}$ is the measured value of a joint meter near the overwintering layer.

It can be seen from Fig. 12; Tables 4, 5 that the temperature change factor proposed in this paper also belongs to the category of temperature component, showing small fluctuations on the curve, and the temperature component curve is no longer smooth, which can accurately reflect the effect of severe temperature changes in cold regions on concrete dam deformation. The addition of freeze-thaw factors reflects the influence of freeze-thaw on the deformation of concrete dams in cold regions. Because the location of the measuring point is far from the wintering layer, compared with other components, the wintering layer component has less influence in this calculation example, which is in line with the actual engineering.

In conclusion, considering the effects of temperature change, freeze-thaw and overwintering layers under special working conditions in cold regions, the monitoring sequence characterization model established has high accuracy and strong applicability, which can better describe the structural behavior changes of concrete dams in cold regions, and can objectively quantify the effects of various influencing factors.

Similarly, other monitoring sequence characterization models such as seepage, stress and strain can be established accordingly, which will not be repeated in the research.

## Conclusions

The paper studies the variation characteristics of the monitoring sequences of concrete dams in cold regions under the joint effect of complex environment and long-term loads, comprehensively reflects the effects of cold waves, freeze-thaw and overwintering layers, and proposes a characterization method for the monitoring sequences. The major results and conclusions are as described below:

1. Regarding the gross error and missing data in the monitoring sequences, the VMD method is used to decompose it into different modal component sub-sequences, which effectively solves the problems of modal aliasing and end-point effects. Based on this, a method for identifying gross errors in monitoring sequence is proposed. Based on the GRU neural network, a missing data processing method based on deep learning is further proposed, which realizes the processing of continuous missing data.
2. On the basis of the insufficient research on the characterization methods of traditional concrete dam structural behavior, in order to fully reflect the effects of cold wave, freeze-thaw and overwintering layer, influencing factors are constructed separately. The expression provides a new method for the objective quantitative analysis of the effect of each influencing factor.
3. In order to describe the spatial distribution characteristics of the monitoring sequences and reflect the correlation between the measured values, the spatial coordinate variables are introduced to establish a characterization model for the change of the monitoring sequences of concrete dams in cold regions, thus realizing the characterization of the overall change law of the monitoring sequences. The validity of the established model is verified through the application of practical engineering.

This method proposed in the manuscript will be further applied to more dams in Northeast and Northwest China, and the model will be optimized based on application feedback. In addition to the above research analysis, the impact of possible cracking and reinforcement measures on the operation status of the project can be analyzed in the future research. In the following work, we can accurately simulate dam cracks in the finite element model, calculate the temperature field, and fit it with the temperature field measured by the actual thermometer. We should also pay attention to the changes in the calculation parameters of the structural parts before and after reinforcement, in order to further improve the characterization model and enhance its applicability.

## Data Availability

The data that support the findings of this study are available on request from the corresponding author upon reasonable request.
